# Differential effects of Huang-Lian-Jie-Du Decoction on Alzheimer’s disease and normal rats

**DOI:** 10.3389/fphar.2025.1710919

**Published:** 2025-11-11

**Authors:** Manru Xu, Yue Zhu, Jinxin Chen, Furong Zhong, Ruoli Wang, Jie Li, Mengyuan Qiao, Yiran Fan, Pan Ren, Mingqi Chen, Jingbo Qin, Wenbin Wu

**Affiliations:** 1 Hospital of Chengdu University of Traditional Chinese Medicine, Chengdu, China; 2 Key Laboratory of Visual Function and Ophthalmopathy, Chengdu, China

**Keywords:** HLJDD, AD, Chinese medicine compound complex, metabolomics, traditional Chinese medicine

## Abstract

**Introduction:**

Huang-Lian-Jie-Du Decoction (HLJDD), a botanical drug used in traditional medicine, has been used in the management of Alzheimer’s disease (AD). However, the mechanisms underlying its preventive effects remain inadequately understood, particularly due to the absence of metabolomic studies examining alterations in serum and cerebrospinal fluid (CSF) metabolites. Moreover, the potential toxicities and side effects of HLJDD necessitate further pharmacological investigation. This study aims to explore the differential effects of HLJDD on AD model rats and healthy controls through a metabolomics approach and uncover the underlying mechanisms based on changes in serum and CSF metabolites. The findings are expected to provide a scientific foundation for enhancing the clinical safety and rational use of HLJDD.

**Methods:**

The composition of HLJDD was characterized by UPLC-Q-Exactive Orbitrap HRMS. Aβ_1-42_-induced SD rats served as the AD animal model. Rats in the sham + HLJDD and Aβ_1-42_ + HLJDD groups (0.604 g/kg freeze-dried powder) were treated with HLJDD via gavage for 28 days. Nissl staining was performed to assess hippocampal neuronal changes, while H&E staining was used to evaluate histopathological alterations in the brain, liver, kidneys, stomach, large intestine, and small intestine. Aβ expression was determined using IHC and ELISA, and inflammatory levels in both peripheral and central systems were quantified by ELISA. MMP-2 and MMP-9 expression were analyzed through IHC. LC-MS was employed to investigate metabolic variations in serum and CSF.

**Results:**

HLJDD reduced Aβ deposition in Alzheimer’s disease rats, enhanced neuronal survival, reduced inflammation, preserved blood-brain barrier (BBB) integrity, and alleviated damage to the brain, kidneys, and stomach. These therapeutic effects were associated with the arginine biosynthesis pathway and ferroptosis. In contrast, HLJDD induced peripheral and central inflammation, impaired neuronal function, compromised BBB integrity, and caused damage to the liver, kidneys, and large intestine in normal rats. These adverse effects were linked to disruptions in aminobenzoate degradation and nucleotide metabolism.

**Conclusion:**

HLJDD may alleviate Aβ-induced damage repair in Alzheimer’s disease rats, but it also induces varying degrees of toxicity in normal rats.

## 1 Introduction

Alzheimer’s disease (AD) is a progressive neurological disorder characterized by memory loss and cognitive impairments (Blanchard et al., 2022). Recent research has focused on the pathogenesis of AD, with Amyloid-beta (Aβ)-mediated neuroinflammation identified as a central mechanism in its development (Alam et al., 2016; Finneran and Nash, 2019; Huang and Mucke, 2012; Leng and Edison, 2021). Soluble Aβ oligomers (AβOs) correlate more strongly with the severity of cognitive decline than other Aβ species. The neurotoxic effects of Aβ aggregates, particularly AβOs, within the brain have been widely acknowledged (Haass and Selkoe, 2007). Inflammatory cytokine overexpression promotes Aβ deposition, leading to neuronal damage and synaptic loss (Gehrmann et al., 1995). This inflammatory environment significantly compromises the blood-brain barrier (BBB), exacerbating the inflammatory response and influencing AD progression (Halliday et al., 2016; Poole et al., 2014). Despite the approval of Aβ-targeted drugs by the U.S. Food and Drug Administration (FDA), their high cost, adverse effects, and contraindications hinder their clinical application and widespread use (Hollmann, 2022).

Huang-Lian-Jie-Du Decoction (HLJDD), a traditional herbal formulation consisting of *Rhizoma Coptidis*, *Radix Scutellariae, Cortex Phellodendri*, and *Fructus Gardeniae*, originated in the Zhou-Hou-Bei-Ji-Fang (Emergency Prescriptions Handbook, circa 4th century CE), as outlined in classical pharmacopeia (Jy et al., 2023). Its long-standing clinical relevance is reflected in its inclusion in both Qi-Xiao-Liang-Fang (1470 CE) and Wai-Tai-Mi-Yao (752 CE), continuing to be used in Traditional Chinese Medicine (TCM) for over eighteen centuries, primarily for heat-clearing and detoxifying purposes (X. Yang et al., 2024). Modern research has expanded its pharmacological scope, demonstrating potential therapeutic benefits in metabolic disorders, including tumors, type 2 diabetes, and neurodegenerative diseases like AD (Durairajan et al., 2014; Wang et al., 2015; X.-J. Zhang et al., 2014). In recent years, HLJDD has been employed in the treatment of AD in China and other Asian countries (Fang et al., 2013; Okamoto et al., 2013). HLJDD can modulate gut microbiota dysbiosis, reduce Aβ accumulation, and alleviate cognitive dysfunction (X et al., 2021; Zheng et al., 2023). Furthermore, clinical trials have shown that HLJDD, when combined with donepezil, enhances cognitive function in patients with AD and potentiates the anti-inflammatory effects of donepezil (Xu et al., 2023). However, the precise mechanisms underlying HLJDD’s effects on AD remain unclear. Currently, most research has predominantly focused on specific mechanistic pathways, lacking a global profile of metabolic changes in serum and cerebrospinal fluid (CSF). To capture the holistic and systemic mechanisms of this multi-component herbal medicine, we adopted a metabolomics approach, which provides insights beyond targeted analysis and may elucidate novel mechanisms of action. With the increasing clinical use of traditional Chinese medicine (TCM), reports of adverse reactions have also risen. Yamano et al. suggested that metabolites of Gardenia components could be linked to hepatotoxicity (Yamano et al., 1988; Yamano et al., 1990), while other studies propose that Gardenia may reduce hepatotoxicity in rats (L et al., 2017) and exhibit anti-inflammatory and antioxidant properties (Shin et al., 2021). This raises questions regarding its hepatoprotective versus hepatotoxic effects. Coptis chinensis, a bitter, cold-natured botanical drug containing berberine, is known to bind to bitter taste receptors (TAS2Rs). In individuals sensitive to these receptors, this interaction may induce vomiting and diarrhea (Avau et al., 2015; Meyerhof et al., 2010; Yue et al., 2018). The Medical Classic of the Yellow Emperor asserts that when therapeutic interventions are precisely tailored to a patient’s pathophysiological profile and accurately implemented, even pharmacologically toxic substances can be utilized safely (Peng et al., 2015). Therefore, the toxicological evaluation of Chinese materia medica requires a comprehensive approach that integrates the recipient’s constitutional traits and current homeostatic conditions. Presently, most toxicological studies of TCM focus on organ damage induced by single botanical drugs, which does not align with the clinical application of botanical drug formulations (Lu et al., 2020). This approach neglects the differential effects of multiple components in botanical drug formulations under pathological and physiological conditions, limiting a full understanding of HLJDD’s scientific basis and its clinical relevance. This gap underscores the rationale for including a healthy control group in our study. Comparing the effects of HLJDD on AD rats versus normal rats is essential to determine whether its therapeutic and adverse effects are disease-state specific. This design enables the distinction between genuine disease-modifying effects and general physiological impacts, thereby critically informing its safety profile and appropriate clinical application for AD patients.

In this study, the differential effects of HLJDD in AD and normal rats were examined. HLJDD was administered orally at a dose of 3 g/kg (equivalent to the crude drug), which was determined to be the optimal concentration based on our previous research. Since the primary aim was to compare the effects of HLJDD between the two groups, only this single dose was used in the experiments. In this study, we investigated the differential effects of HLJDD in AD and normal rats were examined. A single oral dose of 3 g/kg (crude drug) HLJDD was used. The selection of this dose was based on a systematic dose-response investigation in our previous work (Dong et al., 2021), which tested 1.5, 3, and 6 g/kg (crude drug). The 3 g/kg dose was determined to be optimal as it consistently produced significant improvements in learning and memory behaviors and effectively downregulated key molecules of the hippocampal NLRP3/Caspase-1/IL-1β pathway. Its efficacy was comparable to the 6 g/kg dose across most key indicators, making it the most cost-effective choice. As the primary aim of the present study was to compare the effects of HLJDD between groups rather than to re-establish dose dependency, this single, efficacious dose was deemed appropriate.

Pathological changes in AD were assessed through H&E staining and Nissl staining of brain tissue, immunohistochemistry (IHC), and enzyme-linked immunosorbent assay (ELISA) for Aβ detection. Peripheral and central inflammatory cytokine levels, including tumor necrosis factor-alpha (TNF-α), interleukin-1β (IL-1β), and interleukin-4 (IL-4), were measured via ELISA in serum and CSF. BBB integrity was evaluated through IHC detection of MMP-2 and MMP-9. Pathological changes in the liver, kidneys, stomach, large intestine, and small intestine were observed via H&E staining. Additionally, metabolic profiles in serum and CSF were analyzed. By characterizing the distinct impacts of HLJDD on rats in different physiological states, this study offers valuable insights into its potential rational clinical application for AD treatment and strategies to enhance its clinical safety.

## 2 Materials and methods

### 2.1 Chemical regents

*Rhizoma Coptidis*, *Radix Scutellariae*, *Cortex Phellodendri*, and *Fructus Gardeniae* were sourced from Sichuan Neautus Traditional Chinese Medicine Co., Ltd. (China, production batch numbers: 2207067, 2205041, 2208121, 2208046). β-Amyloid 1-42 (Aβ_1-42_ oligomer) was obtained from Med Chem Express (United States, production batch number: HY-P1388). Huperzine A was purchased from Shanghai Macklin Biochemical Technology Co., Ltd. (China, production batch number: 102518-79-6). The IHC antibodies for MMP-2 and MMP-9 were sourced from ZEN-BIOSCIENCE (China, production batch number: R380817, 1:50) and Servicobio (China, production batch number: GB11132, 1:100), respectively. The Aβ antibody was obtained from Proteintech (China, production batch number: 25524-1-AP, 1:200). The ELISA kits for TNF-α, IL-1β, and IL-4 were purchased from JINGMEI (China, TNF-α: production batch number: JM-01587R1; IL-1β: production batch number: JM-01454R1; IL-4: production batch number: JM-01598R1). The Aβ_42_ ELISA kit was obtained from Fine (China, production batch number: ER0755).

### 2.2 Preparation and quality control of Huang-Lian-Jie-Du decoction

The HLJDD formulation was prepared using a specific ratio of its components: *Rhizoma Coptidis*, *Radix Scutellariae*, *Cortex Phellodendri*, and *Fructus Gardeniae* in a dry weight ratio of 9:6:6:9 (Table 1). All botanical drugs were purchased from Sichuan Neautus Traditional Chinese Medicine Co., Ltd., and authenticated as genuine by Professor Yuntong Ma from the College of Pharmacy, Chengdu University of Traditional Chinese Medicine. The botanical drugs were thoroughly mixed, and water was added in successive volumes of 10, 8, and 8 times the dry botanical drug weight, respectively. The mixture was soaked for 30 min and then extracted three times at 120 °C. The extracts were stored at 4 °C for preservation. After two rounds of extraction, the combined liquids were concentrated using a rotary evaporator under reduced pressure and then freeze-dried. Regarding the dosage, the human clinical dose was set at 30 g of crude drug per day for a 60-kg adult. Based on established allometric scaling principles, the equivalent dosage for rats was calculated to be 6 times that of humans, resulting in 3 g/kg/day of crude drug. Considering the yield of the lyophilized powder was 20.13%, the final administered dose was determined as 0.604 g/kg, prepared at a concentration of 0.06 g/mL.

**TABLE 1:** Composition of HLJDD.

Chinese name	Pharmaceutical name	Plant name	Weight(g)
Huanglian	Coptidis rhizoma	*Coptis chinensis Franch*	9
Huangqin	Scutellariae radix	*Scutellaria baicalensis Georgi*	6
Huangbai	Phellodendri chinensis cortex	*Phellodendron Chinense Schneid*	6
Zhizi	Gardenia fructus	*Gardenia jasminoides Ellis*	9

The HLJDD powder was dissolved in 80% methanol, sonicated, and mixed for 40 min, then passed through a 0.22 μm microporous filter membrane for detection. The final analysis was conducted using UPLC-Q-Exactive Orbitrap HRMS. The experiments were performed on a Thermo Scientific UPLC system (Waltham, MA, United States) coupled with high-resolution mass spectrometry (HRMS, Q Exactive Orbitrap). Chromatographic separation was carried out on an Ultimate UHPLC XB-C18 column (2.1 mm × 100 mm, 1.8 µm). Data were collected in full-scan mode (Full-MS), with a scanning range of m/z 100–1,500 Da (resolution: 35,000). A secondary scan (dd-MS2, m/z 100–1,500 Da, resolution: 17,500) was also employed.

### 2.3 Experimental animals

Male Sprague-Dawley (SD) rats (specific pathogen-free [SPF] grade, 8 weeks old, weighing 200 ± 20 g) were obtained from Chengdu DOSSY Experimental Animal Co., Ltd. The animals were housed in the laboratory of the School of Pharmacy at Chengdu University of Traditional Chinese Medicine, under license number SYXK (Sichuan) 2020-030. Rats were acclimatized for 1 week at a temperature of 22 °C ± 1 °C and relative humidity of 55% ± 5%. Each rat was provided with 30 g of standard feed daily and had *ad libitum* access to water.

In this study, a total of 30 rats were randomly allocated into five groups (*n =* 6 per group) as follows: the Sham group, the Aβ_1-42_ group, the Huperzine A (Hup A) + Aβ_1-42_ group, the Sham + HLJDD group, and the HLJDD + Aβ_1-42_ group. The randomization was performed using a random number table generated by Microsoft Excel, where each animal was assigned a number and then allocated to one of the groups based on the numerical order to ensure an unbiased grouping procedure. Regarding the interventions, the Sham and Sham + HLJDD groups received bilateral injections of 5 μL of control solvent into the hippocampal CA1 region, while the other three groups were administered 5 μL of Aβ_1-42_ bilaterally into the same region to establish the model. Aβ_1-42_ oligomer (5 mg) was dissolved in 1,130 μL of Hexafluoroisopropanol (HFIP) to create a uniform solution, which was incubated. Then, DMSO and PBS were added to reach a final concentration of 4 μg/μL. The solution was incubated at 4 °C for 24 h. For the control solvent, Aβ_1-42_ oligomers were omitted, with all other conditions remaining identical. After a 7-day recovery period, all groups received continuous gavage for 28 days.

The gavage doses for rats were calculated based on human equivalent doses adjusted for body surface area: HLJDD 0.604 g/kg freeze-dried powder (equivalent to 3 g/kg crude drug; yield 20.13%) and Hup A 2 × 10^−5^ g/kg. Control rats were given equivalent ultrapure water, with each group receiving 1 mL/100 g by gavage. The body weight of the rats was recorded (in grams) every 7 days. On the 28th day of the experiment, the rats were euthanized via intraperitoneal injection of 2% pentobarbital sodium 1 h after gavage. Blood samples were collected via abdominal aorta puncture, and CSF, brain, liver, kidney, large intestine, small intestine, and stomach tissues were harvested for subsequent analysis. All animal experiments were conducted in strict accordance with relevant laws and ethical guidelines and were approved by the Animal Ethics Committee of Chengdu University of Traditional Chinese Medicine (Approval No.: 2024DL-018; Date of approval: 3 June 2024).

### 2.4 Histopathological analysis

Tissues from the brain, liver, kidney, large intestine, small intestine, and stomach were fixed, embedded, and sectioned. Dewaxing and hydration were performed, followed by H&E staining. Brain tissue underwent both H&E and Nissl staining, and neutral resin adhesive was used for transparent sealing. Morphological and structural changes in the tissues were observed under a light microscope.

### 2.5 Measurement of cytokines

Blood and CSF (cisterna magna) were collected from each rat, centrifuged at 4 °C, 3,000 rpm for 10 min. All procedures were carried out according to the kit instructions. The control was diluted to the specified multiplicity as instructed. Seven concentration series were established for each inflammatory factor (IL-4, TNF-α, IL-1β) and Aβ42 to prepare the standard curves, with the corresponding concentrations determined based on the optical density (OD) values of the samples.

### 2.6 Immunohistochemical analysis

Brain tissues were fixed in paraformaldehyde solution, dehydrated with alcohol, subjected to xylene transparency, and embedded using a three-step dip-waxing method, followed by sectioning. The slices were baked in an oven for routine dewaxing, rinsed with PBS three times, treated with 3% H_2_O_2_ for 10 min, and rinsed again with PBS three times. Antigen retrieval was performed using a microwave method, followed by additional PBS rinsing. The PBS was blotted dry, and the tissue sections were circled with an IHC pen. Bovine serum blocking solution was added for 20 min at room temperature. Primary antibodies against MMP-2 (1:50), MMP-9 (1:100), and Aβ_42_ (1:200) were applied and incubated in a wet box at 4 °C overnight. The sections were rinsed with PBS, and DAB was used for color development at room temperature. Hematoxylin was used for nuclear counterstaining, followed by rinsing with running water. The sections were then dehydrated, mounted, and images were captured using a microcamera for analysis and counting.

### 2.7 Detection of serum and CSF metabolites using liquid chromatography mass spectrometer (LC-MS)

For LC-MS analysis, 100 μL of serum and 100 μL of CSF were mixed with 400 μL of extraction solution, vortexed for 30 s, and extracted using low-temperature ultrasonic extraction. The supernatant was transferred to an injection vial for online analysis after re-solution. Additionally, 20 μL of each sample was mixed separately as quality control (QC) samples. LC-MS was performed using a UHPLC-Q Exactive HF-X system. Chromatographic conditions included an ACQUITY UPLC HSS T3 column (100 mm × 2.1 mm i.d., 1.8 µm; Waters, Milford, United States of America), with mobile phase A consisting of 95% water and 5% acetonitrile (containing 0.1% formic acid) and mobile phase B consisting of 47.5% acetonitrile, 47.5% isopropanol, and 5% water (containing 0.1% formic acid). The injection volume was 3 μL, and the column temperature was set to 40 °C.

### 2.8 Statistical analysis

Statistical analyses were performed using SPSS 26.0, and data were graphed using GraphPad Prism 7 software. One-way analysis of variance (ANOVA) followed by LSD *post hoc* tests was applied to all dependent variables between groups. Data were expressed as the mean ± standard deviation (SD), with *P*-values <0.05 considered significant. The value of n refers to the number of rats. Raw data generated by UPLC-Q-Exactive Orbitrap HRMS were processed using Xcalibur software (version 3.1) for relative quantification. Metabolomics analysis was conducted using ProgenesisQI software (Waters Corporation, Milford, United States of America).

## 3 Results

### 3.1 UPLC-Q-exactive orbitrap HRMS analysis identified the primary chemical metabolites of HLJDD

**FIGURE 1:**
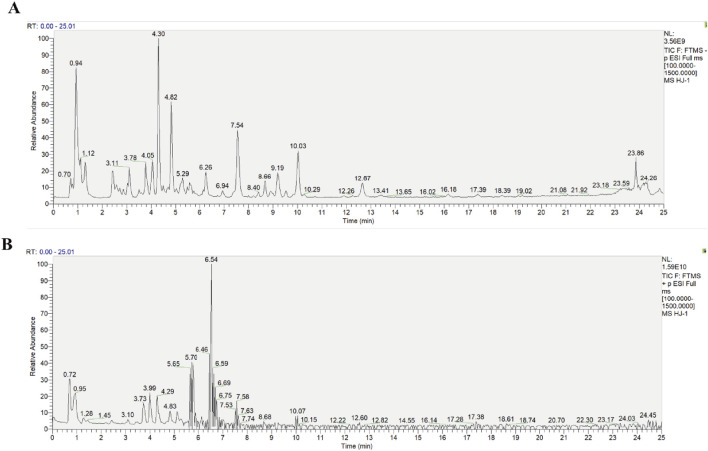
Diagram of the total ion flow of the traditional Chinese medicine metabolite Huang-Lian-Jie-Du Decoction in ion modes. **(A)** Negative ion mode, **(B)** Positive ion mode.

The primary chemical metabolites of HLJDD were identified using UPLC-Q-Exactive Orbitrap HRMS. The total ion flow diagram of HLJDD in both negative and positive ion modes is shown in Figure 1. A total of 23 metabolites, including Neochlorogenic acid, Geniposide, Berberine, Scutellarin, and Baicalin, were identified. Detailed information about these metabolites is provided in Table 2.

**TABLE 2:** Chemical composition of Huang-Lian-Jie-Du Decoction identified using UPLC-Q-Exactive Orbitrap HRMS.

No.	Formula	tR/min	Theoretical (Da)	Calculated mass (Da)	Error (ppm)	Adducts	MS fragmentation	Component name	mzCloud best match	mzVault best match
1	C_16_H_18_O_9_	3.19	355.09528	355.10288	0.56	[M+H]^+1^	191.05556, 179.03435, 135.04431	Neochlorogenic acid	95.9	94.3
2	C_16_H_18_O_9_	3.84	353.09515	353.08781	0.23	[M-H]^−1^	191.05573	Chlorogenic acid	99.9	95.1
3	C_20_H_23_NO_4_	3.99	342.16324	342.17055	1.57	[M+H]^+1^	326.10208, 299.13851	Phellodendrine	98.7	92.3
4	C_17_H_24_O_10_	4.29	433.13667	433.13481	−0.72	[M-H]^−1^	225.07531, 175.03895, 147.04342	Geniposide	98.8	92.6
5	C_9_H_8_O_4_	4.34	179.04162	179.03435	−3.52	[M-H]^−1^	135.04437, 113.11392	Caffeic acid	99.8	97.4
6	C_7_H_12_O_6_	4.82	191.06269	191.05542	−3.63	[M-H]^−1^	173.00813, 85.02856	Quinic acid	94.2	86
7	C_10_H_10_O_4_	4.84	195.05806	195.06534	0.81	[M+H]^+1^	178.90244, 134.59469	Ferulic acid	98.6	93.4
8	C_19_H_15_NO_4_	5.11	322.10665	322.10794	1.73	[M+H]^+1^	307.08389, 279.08911	Berberrubine	93.9	85.9
9	C_27_H_30_O_16_	5.24	609.15372	609.14632	0.56	[M-H]^−1^	365.69574, 300.02716, 243.78491	Rutin	99.7	95.9
10	C_26_H_34_O_11_	5.35	521.20019	521.20302	0.14	[M-H]^−1^	359.14886, 329.13980	Lariciresinol 4-O-glucoside	94.8	86.1
11	C_19_H_13_NO_4_	5.79	320.08461	320.09189	0.37	[M+H]^+1^	292.09695, 262.08643	Coptisine	92.2	86.5
12	C_20_H_20_NO_4_	5.80	338.13125	338.13853	18.89	[M+H]^+1^	322.10773, 308.09183, 294.11264	Jatrorrhizine	93.8	92.3
13	C_44_H_64_O_24_	6.26	975.37895	975.37116	0.21	[M-H]^−1^	327.15983, 283.17015, 239.18008	Crocin	87.7	85.8
14	C_20_H_17_NO_4_	6.57	336.1163	336.12357	1.61	[M+H]^+1^	320.09198, 292.09706, 306.07642	Berberine	97.1	94.7
15	C_21_H_22_NO_4_	6.95	352.14715	352.15443	0.43	[M+H]^+1^	336.12326, 308.12823, 322.10767	Palmatine	-	92.2
16	C_21_H_18_O_12_	7.55	461.06975	461.07278	−0.16	[M-H]^−1^	461.07261, 285.04053	Scutellarin	97.4	89.2
17	C_21_H_18_O_11_	7.56	447.09494	447.0924	0.07	[M+H]^+1^	269.04667	Baicalin	99.8	94.9
18	C_16_H_12_O_5_	8.39	283.06168	283.06116	0.73	[M-H]^−1^	285.07599, 270.05310	Glycitein	95.6	82.9
19	C_22_H_20_O_11_	8.46	461.10804	461.10853	1.04	[M+H]^+1^	269.04565	Baicalin methyl ester	-	85.9
20	C_21_H_18_O_11_	8.99	445.07519	445.07774	0.63	[M-H]^−1^	445.07115, 269.04641	Apigenin 7-O-glucuronide	99.5	83.5
21	C_22_H_20_O_11_	10.02	459.10051	459.09317	−0.12	[M-H]^−1^	283.06312, 268.03921	Wogonoside	-	92.5
22	C_15_H_10_O_5_	12.69	269.04297	269.04558	0.55	[M-H]-1	269.04535, 241.04561, 223.02674	Baicalein	99.6	92.4
23	C_16_H_12_O_5_	17.38	285.07869	285.07598	0.77	[M+H]^+1^	270.05065	Wogonin	99.8	93.4

Data were collected in full-scan mode (Full-MS) with a scanning range of m/z 100–1,500 Da (resolution: 35,000); the data were further analyzed with a secondary scan (dd-MS2, m/z 100–1,500 Da, resolution 17,500).

### 3.2 Effect of HLJDD on pathological damage in the brain of the Alzheimer’s disease and normal rats

**FIGURE 2:**
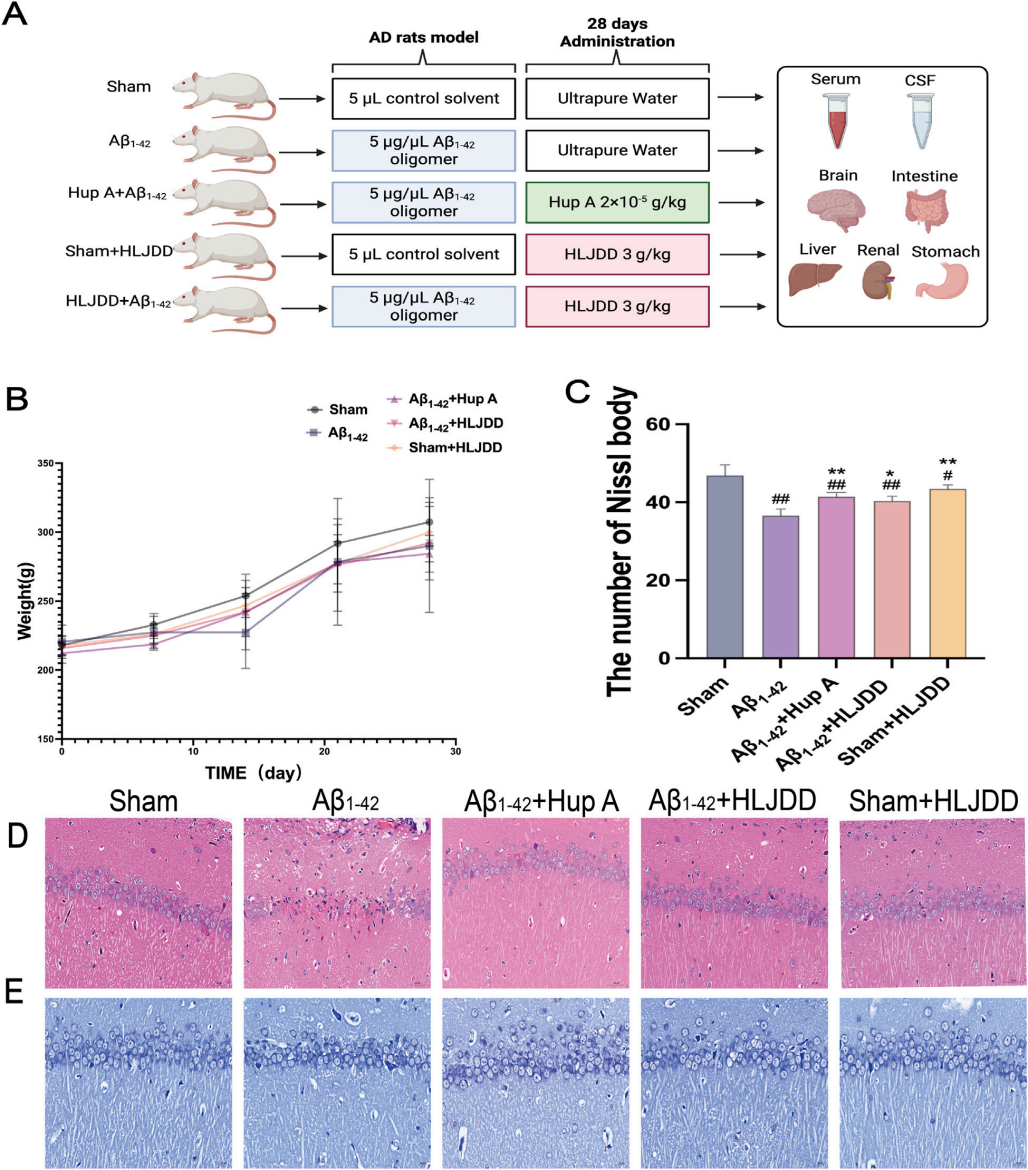
Effect of HLJDD on pathological damage in the brains of Alzheimer’s disease and normal rats. **(A)** Experimental design, **(B)** Body weight of rats (*n =* 6), **(C)** Number of Nissl bodies (*n =* 3), **(D)** H&E staining (Magnification ×400, scale bars: 50 μm) (*n =* 3), **(E)** Nissl staining (Magnification ×400, scale bars: 20 μm) (*n =* 3). Data are presented as means ± SD. ^#^ *P* < 0.05, ^##^ *P* < 0.01 vs. Sham group; ^*^ *P* < 0.05, ^**^ *P* < 0.01 vs. Aβ_1-42_ group.

The experimental timeline is presented in Figure 1A. Throughout the study, qualitative observations were recorded. Sham-operated rats displayed a sleek coat, normal posture and gait, and formed, solid feces. In contrast, rats in the Aβ_1-42_ model group presented with a ruffled and unkempt coat, a hunched posture, decreased spontaneous activity, and loose stools. HLJDD treatment ameliorated the appearance of the coat and improved activity levels and general condition in the model groups. Notably, sham-operated rats administered HLJDD exhibited piloerection, signs of agitation in response to handling, perianal soiling, and hard, dry feces. Body weight, as a direct indicator of growth, gradually increased across all groups, with no significant differences observed at the end of the experiment (Figure 2B).

H&E staining revealed that HLJDD administration to the sham group did not significantly affect pyramidal cell pathology in the hippocampus CA1 region. In comparison to the sham group, the Aβ_1-42_ group exhibited degenerative necrosis of pyramidal cells, glial cell proliferation, reduced cell volume, intensified staining, and blurred intracellular structures in the hippocampal CA1 region. The Hup A-treated Aβ_1-42_ group showed less pronounced alterations, including an increase in the number of darkly stained neurons and reduced cell volume with intensified staining. However, HLJDD significantly ameliorated the pathological changes in the HLJDD-treated Aβ_1-42_ group (Figure 2D). Nissl staining was conducted to assess neuronal changes. In the Aβ_1-42_ group, there was a significant reduction in the number of healthy and surviving neurons in the hippocampal CA1 region, resulting in typical neuropathological features such as loss of Nissl bodies and nuclear disappearance, compared to the sham group (*P* = 0.000). In contrast, the HLJDD-treated Aβ_1-42_ group showed improved survival of hippocampal neurons and prevented the loss of neurons in the CA1 area as well as the preservation of Nissl bodies (*P* = 0.019). Interestingly, the HLJDD-treated sham group exhibited a decrease in Nissl bodies (*P* = 0.028) (Figures 2C,E). These results suggest that HLJDD can prevent hippocampal neuronal apoptosis in AD rats but may cause functional impairment to neurons in normal rats, with this damage not being exclusively linked to Aβ.

### 3.3 Effect of HLJDD on Aβ accumulation in Alzheimer’s disease and normal rats

**FIGURE 3:**
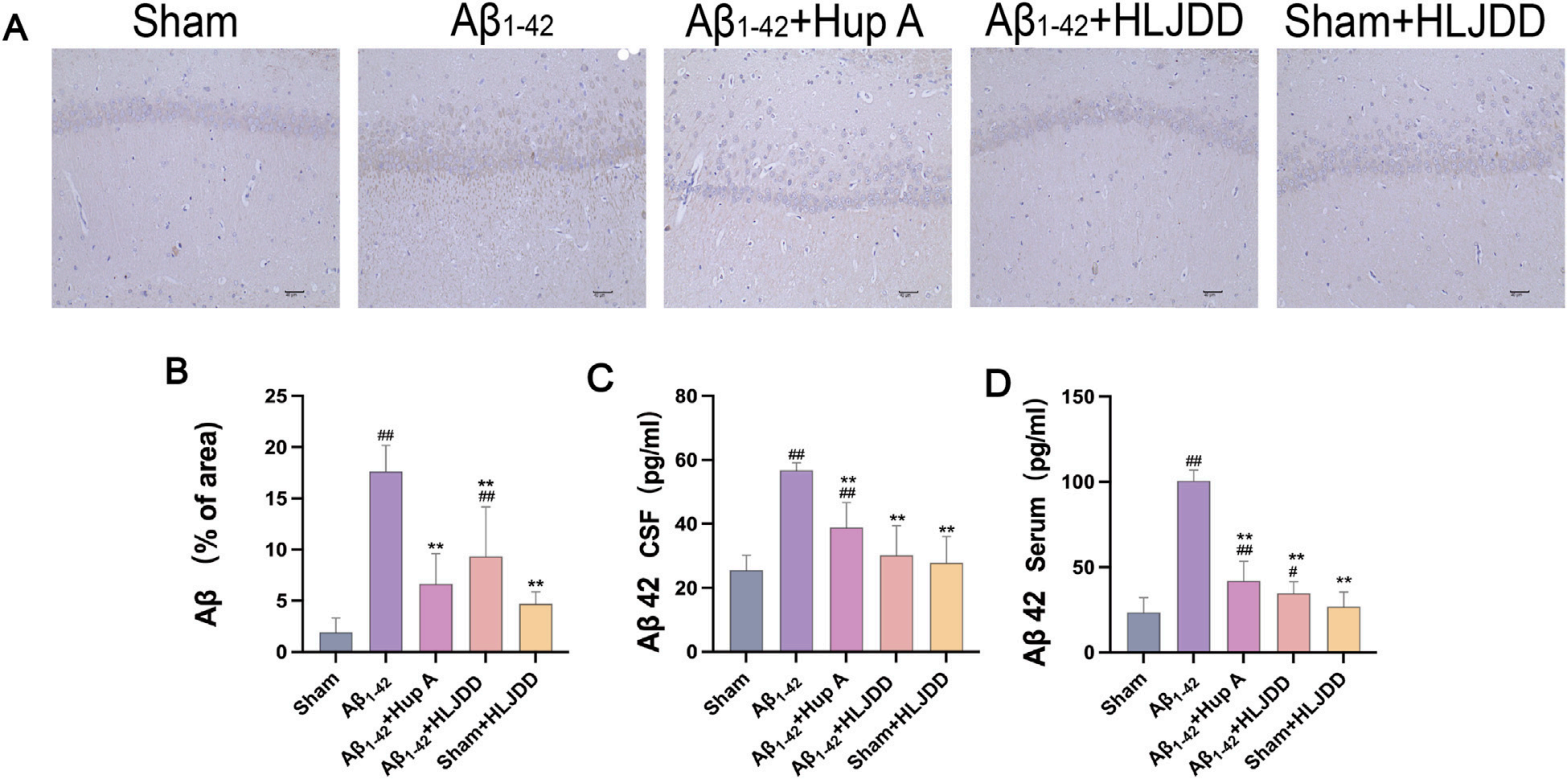
Effect of HLJDD on Aβ accumulation in Alzheimer’s disease and normal rats. **(A)** Immunohistochemical detection of Aβ (Magnification ×200, scale bars: 40 μm) (*n =* 3), **(B)** Immunohistochemical detection of Aβ (% of area) in the CA1 region of the hippocampus (*n =* 3), **(C)** ELISA detection of Aβ levels in CSF (*n =* 6), **(D)** ELISA detection of Aβ levels in serum (*n =* 6). Data are presented as means ± SD. ^#^ *P* < 0.05, ^##^ *P* < 0.01 vs. Sham group; ^*^ *P* < 0.05, ^**^ *P* < 0.01 vs. Aβ_1-42_ group.

Excessive Aβ deposition disrupts the structural integrity of neural synapses (Clinton et al., 2010) and activates pro-inflammatory cytokines leading to neuronal dysfunction (Carrano et al., 2012; LaFerla and Oddo, 2005). To determine whether HLJDD could suppress Aβ accumulation, Aβ expression was assessed through IHC and ELISA. Consistent with the pathological findings, both IHC and ELISA results showed increased Aβ_1-42_ expression in the hippocampal CA1 region, CSF, and serum of the Aβ_1-42_ group (both *P* = 0.000), confirming successful model establishment. Moreover, the expression of Aβ in these regions was significantly reduced in both the Hup A-treated Aβ_1-42_ group (IHC: *P* = 0.001, CSF: *P* = 0.000, serum: *P* = 0.000) and the HLJDD-treated Aβ_1-42_ group (IHC: *P* = 0.006, CSF: *P* = 0.000, serum: *P* = 0.000) (Figure 3). These results indicate that HLJDD exerts neuroprotective effects by reducing Aβ deposition in the brains of AD rats, with no significant impact on Aβ levels in normal rats.

### 3.4 Effect of HLJDD on the inflammation of Alzheimer’s disease and normal rats

**FIGURE 4:**
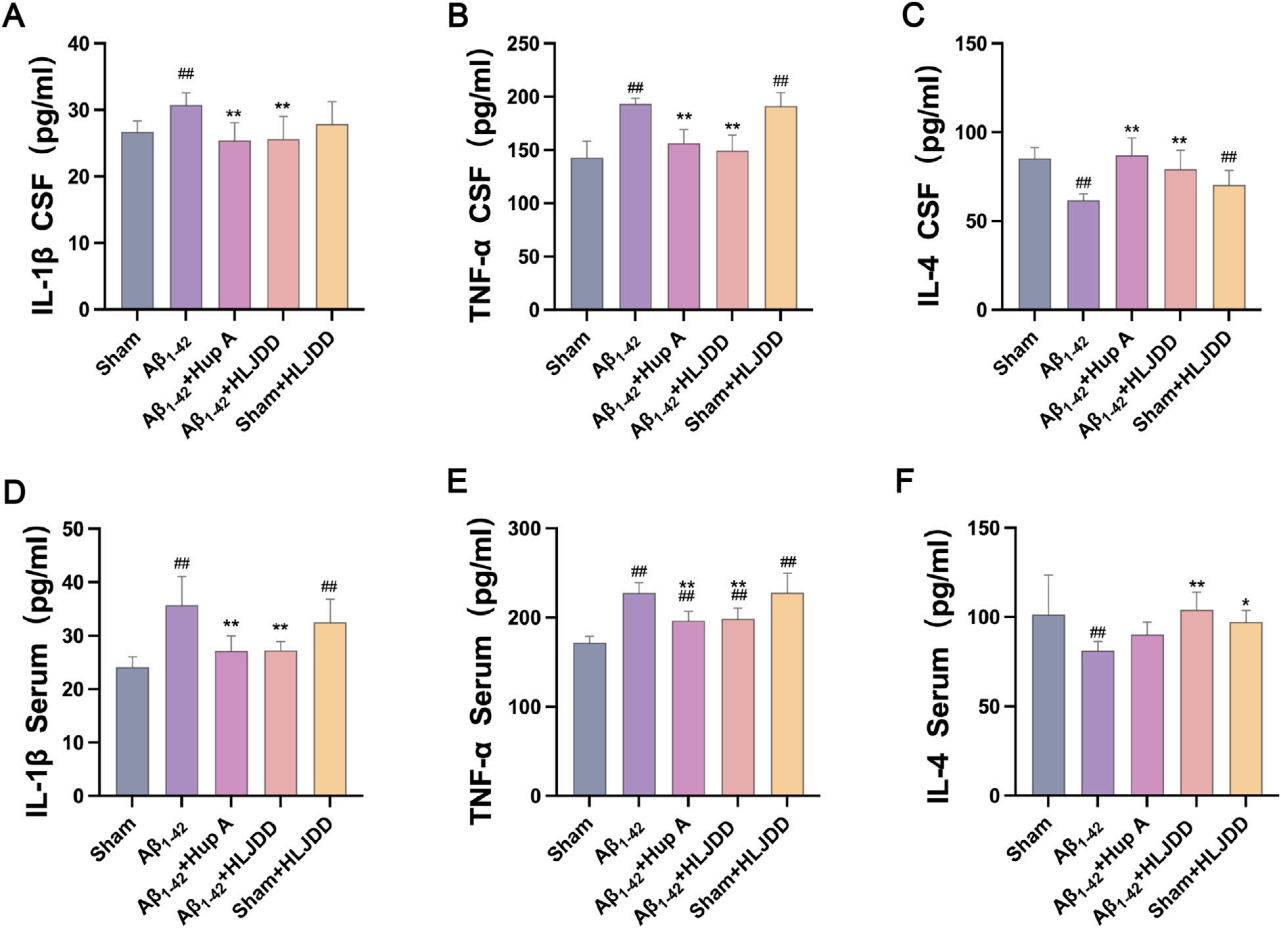
ELISA detection of cytokines (Serum and CSF). **(A)** IL-1β in CSF, **(B)** TNF-α in CSF, **(C)** IL-4 in CSF, **(D)** IL-1β in serum, **(E)** TNF-α in serum, **(F)** IL-4 in serum. Data are presented as means ± SD (*n =* 6). ^#^ *P* < 0.05, ^##^ *P* < 0.01 vs. Sham group; ^*^ *P* < 0.05, ^**^ *P* < 0.01 vs. Aβ_1-42_ group.

This examined the effect of HLJDD on peripheral and central inflammation in both AD and normal rats by measuring IL-1β, TNF-α, and IL-4 in serum and CSF using ELISA kits. The levels of IL-1β and TNF-α were significantly elevated in both the CSF and serum of the Aβ_1-42_ group compared to the sham group, while IL-4 levels were significantly reduced (IL-1β CSF: *P* = 0.015, IL-1β serum: *P* = 0.000, IL-4 CSF: *P* = 0.000, IL-4 serum: *P* = 0.007, TNF-α CSF: *P* = 0.000, TNF-α serum: *P* = 0.000). HLJDD treatment significantly decreased the concentrations of IL-1β and TNF-α in both serum and CSF, while increasing the level of IL-4 (IL-1β CSF: *P* = 0.003, IL-1β serum: *P* = 0.000, IL-4 CSF: *P* = 0.001, IL-4 serum: *P* = 0.003, TNF-α CSF: *P* = 0.000, TNF-α serum: *P* = 0.001). In contrast, compared to the sham group, the HLJDD-treated sham group exhibited significantly higher levels of TNF-α and IL-1β in both serum and CSF, along with a decrease in CSF IL-4 levels, reflecting an inflammatory profile similar to that of the Aβ_1-42_ group (IL-1β serum: *P* = 0.000, IL-4 CSF: *P* = 0.004, TNF-α CSF: *P* = 0.000, TNF-α serum: *P* = 0.000) (Figure 4). These results suggest that while HLJDD alleviates peripheral and central inflammation and offers neuroprotection in AD rats, it may induce peripheral and central inflammation in normal rats.

### 3.5 Effect of HLJDD on the structural integrity of Alzheimer’s disease and normal rats

**FIGURE 5:**
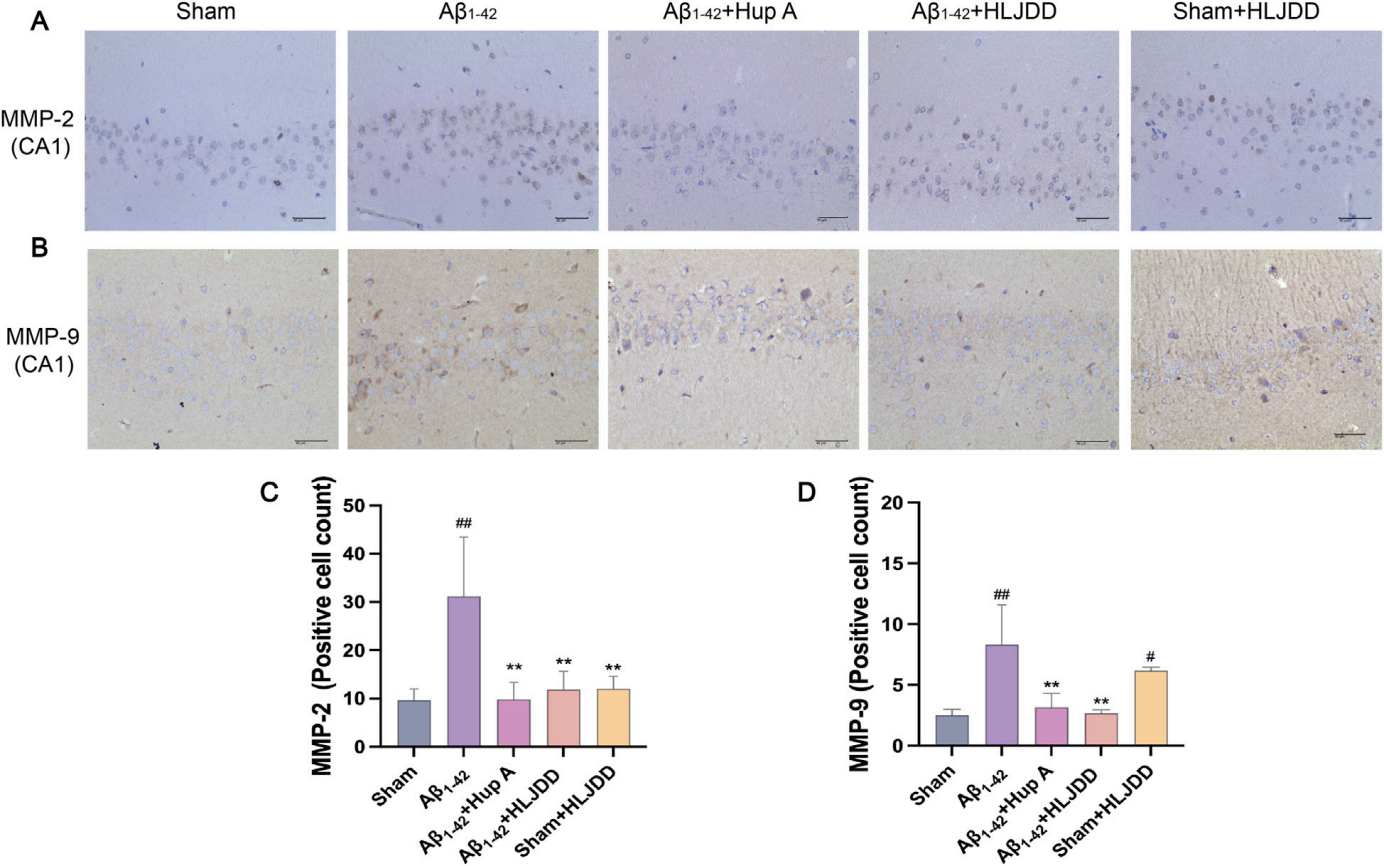
Effect of HLJDD on structural integrity in Alzheimer’s disease and normal rats. **(A,C)** Representative images of MMP-2 staining in the hippocampal CA1 region of brain tissue, and quantification of MMP-2 expression. **(B,D)** Representative images of MMP-9 staining in the hippocampal CA1 region of brain tissue, and quantification of MMP-9 expression. (Magnification ×400, scale bars: 40 μm). Data are presented as means ± SD (*n =* 3). ^#^ *P* < 0.05, ^##^ *P* < 0.01 vs. Sham group; ^*^ *P* < 0.05, ^**^ *P* < 0.01 vs. Aβ_1-42_ group.

Excessive Aβ deposition has been implicated in the disruption of the BBB integrity (Storck and Pietrzik, 2018; van de Haar et al., 2017). MMP-2 and MMP-9 play a role in BBB disruption by degrading basement membrane proteins of cerebral capillaries. To investigate the effect of HLJDD on BBB permeability, IHC analyses were performed to assess MMP-2 and MMP-9 expression in the hippocampal CA1 region of rats (Zhu et al., 2018). Compared to the sham group, the Aβ_1-42_ group exhibited a significant increase in MMP-2 and MMP-9 positive cells (MMP-2: *P* = 0.002, MMP-9: *P* = 0.001). In the HLJDD-treated Aβ_1-42_ group, the expression of MMP-2 and MMP-9 was significantly downregulated (MMP-2: *P* = 0.003, MMP-9: *P* = 0.001). Interestingly, the HLJDD-treated sham group showed an upregulation of MMP-9 (MMP-9: *P* = 0.017) (Figure 5). These preliminary results suggest that while HLJDD can effectively preserve the structural integrity of the BBB in AD rats, it may negatively impact it in normal rats. However, these findings are preliminary and should be interpreted with caution, necessitating further research to validate and elaborate on the observed dual effects.

### 3.6 Effect of HLJDD on the metabolic organs of Alzheimer’s disease and normal rats

**FIGURE 6:**
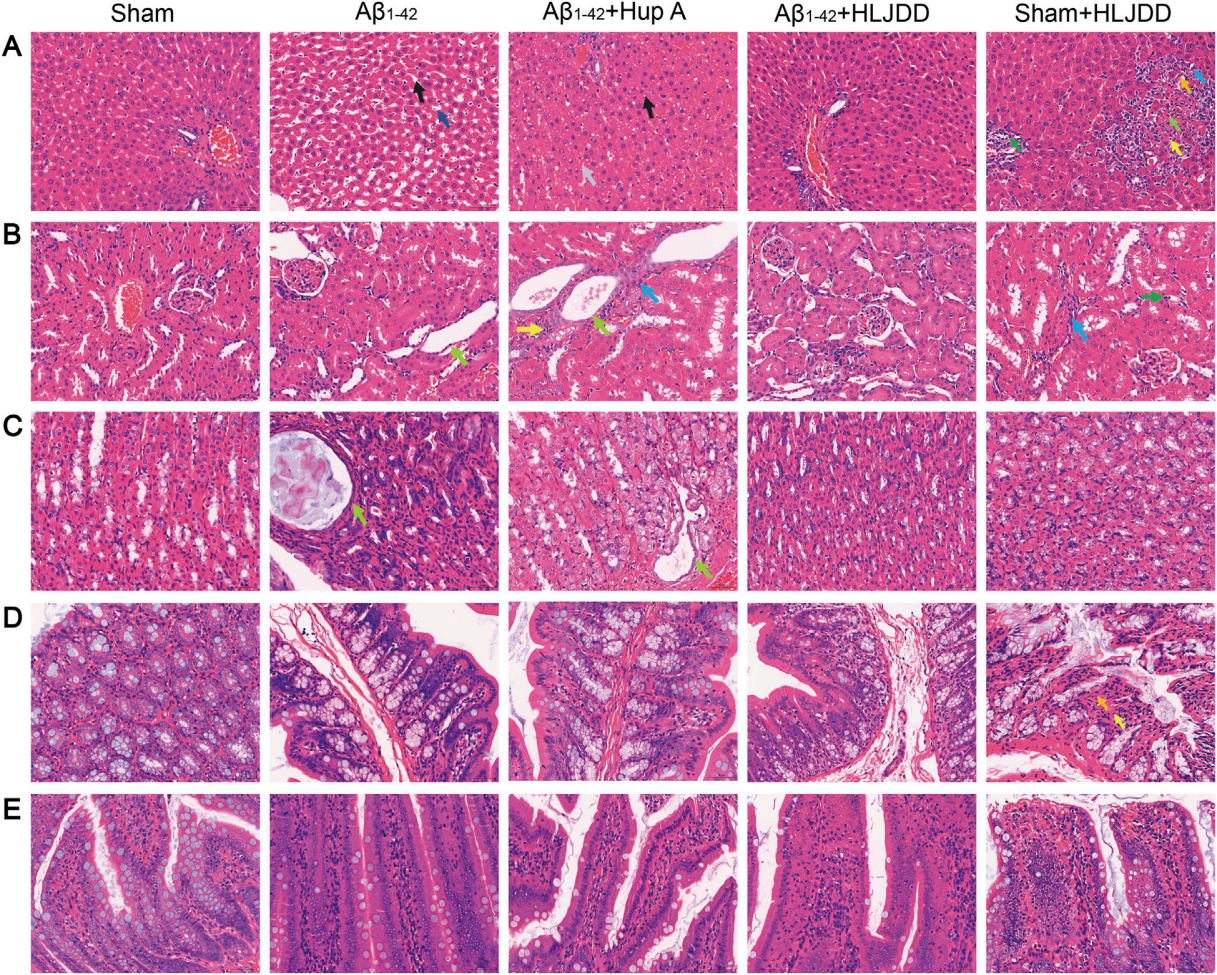
The bidirectional effect of HLJDD on drug-metabolizing organs in Alzheimer’s disease and normal rats. **(A)** Histopathological changes in liver tissues. Hepatocellular necrosis (↑), LymphocyteNeutrophils (↑), Fibroblast (↑), Fibrocyte (↑), Fatty degeneration of liver (↑), Hepatic sinusoidal dilatation (↑), Hepatic sinusoidal congestion (↑). **(B)** Histopathological changes in renal tissues. Renal tubular epithelial cells are flattened (↑), Lymphocyte (↑), Fibrocytes (↑), Neutrophils (↑). **(C)** Histopathological changes in gastric tissues. Gastric glandular epithelial cells are flattened (↑). **(D)** Histopathological changes in large intestine tissues. Lamina propria necrosis (↑), neutrophils (↑). **(E)** Histopathological changes in small intestine tissues. H&E staining of histological sections. (Magnification ×400, scale bars: 50 μm) (*n =* 3).

TCM and its components may pose direct organ-damaging toxicity. To evaluate the potential toxicity of HLJDD, H&E staining was conducted on tissues from the liver, kidneys, stomach, large intestine, and small intestine to assess its effects on the digestive system. In liver tissues, the HLJDD-treated sham group exhibited hepatocellular necrosis, inflammatory cell infiltration, and fibrotic tissue proliferation, while the Aβ_1-42_ and Hup A-treated Aβ_1-42_ groups showed sporadic minor hepatocyte steatosis and vacuolar degeneration. Importantly, HLJDD treatment in the HLJDD-treated Aβ_1-42_ group alleviated these liver pathologies (Figure 6A). In renal tissues, the Aβ_1-42_ group displayed moderate tubular dilatation with flattened tubular epithelial cells, whereas the Hup A-treated Aβ_1-42_ group showed mild interstitial inflammatory infiltration and subtle fibrotic proliferation. While HLJDD treatment attenuated renal lesions in the HLJDD-treated Aβ_1-42_ group, the HLJDD-treated sham group exhibited tubular epithelial swelling, luminal dilatation, proteinaceous casts, inflammatory infiltration, and fibroplasia (Figure 6B). Gastric analysis revealed mild glandular dilatation with epithelial flattening in the Aβ_1-42_ and Hup A-treated Aβ_1-42_ groups, which was mitigated in the HLJDD-treated Aβ_1-42_ group. No gastric abnormalities were observed in the HLJDD-treated sham group (Figure 6C). In colonic tissues, mucosal epithelial detachment, lamina propria necrosis, inflammatory infiltration, and villous dissolution were found exclusively in the HLJDD-treated sham group, with no significant lesions in other groups (Figure 6D). The small intestines in all experimental groups remained structurally intact with no significant histopathological changes (Figure 6E). These results suggest that HLJDD can mitigate pathological damage in the renal and gastric tissues of AD rats. Although serum samples were unavailable for biochemical analysis of indicators such as ALT and AST, histopathological examination clearly revealed tissue injury. Overall, histopathological findings indicated that HLJDD’s toxic effects in normal rats are associated with damage to the liver, kidneys, and large intestine.

### 3.7 Effect of HLJDD on metabolism of Alzheimer’s disease and normal rats analyzed by LC-MS analyses of metabolic profiles

**FIGURE 7:**
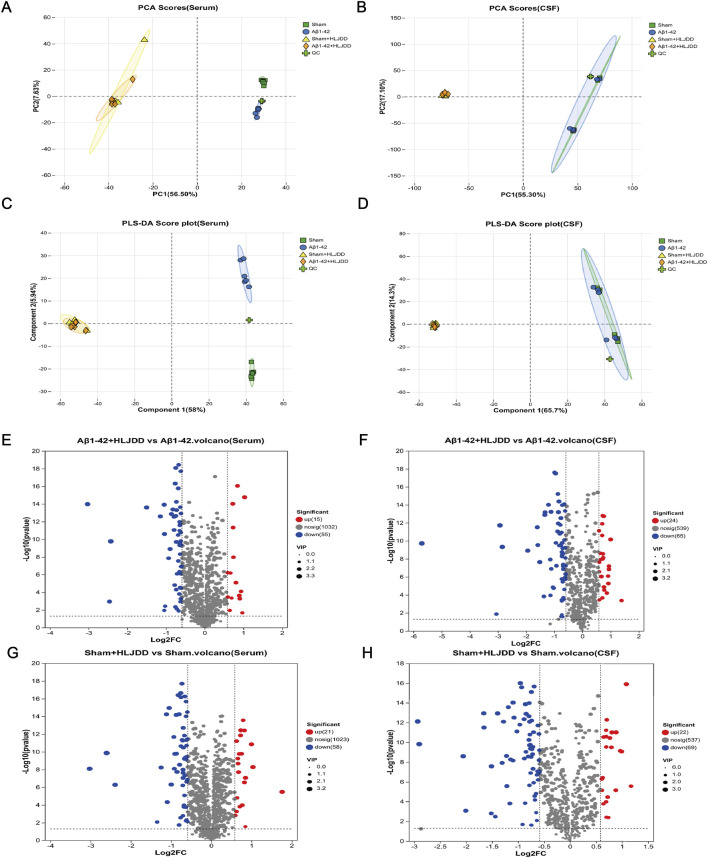
Metabolite analysis of HLJDD in the HLJDD-treated sham group and HLJDD-treated Aβ_1-42_ group rats via untargeted metabolic profiling. **(A)** PCA score (serum), **(B)** PCA score (CSF), **(C)** PLS-DA score (serum), **(D)** PLS-DA score (CSF), **(E)** QC represents a quality control sample. Volcano plot for Aβ_1-42_ + HLJDD vs. Aβ_1-42_ (serum) **(F)** and Aβ_1-42_ + HLJDD vs. Aβ_1-42_ (CSF), **(G)** Sham + HLJDD vs. Sham (serum), **(H)** Sham + HLJDD vs. Sham (CSF). Red: upregulated differential metabolites, blue: downregulated differential metabolites, gray: insignificant differential metabolites. VIP: Variable importance value from the PLS-DA model. The larger the VIP, the greater the contribution of the variable to the grouping (*n =* 6).

Differences in the metabolic microenvironment influence the body’s response to drug treatments. This study examined the variations in HLJDD metabolism in the peripheral and central regions of AD and normal rats using LC-MS analysis on CSF and serum samples. Multivariate analysis, including PCA and PLS-DA, revealed distinct metabolomic profiles among the Sham, Sham + HLJDD, Aβ_1-42_, and Aβ_1-42_ + HLJDD groups. The presence of clusters in the QC samples confirmed the stability of the analytical system, demonstrating good reproducibility and instrument reliability during the analysis (Figures 7A–D). The results indicated the presence of both conserved and differential metabolic constituents under normal and pathological conditions. Using a t-test (*P* < 0.05) and VIP >1, with a fold change threshold of 1.5, differential metabolites between the groups were identified. Comparative metabolomic analysis revealed significant alterations in metabolite profiles across experimental groups. In serum, 70 differentially expressed metabolites (DEMs) were identified between the HLJDD-treated Aβ_1-42_ group and the Aβ_1-42_ group, with 15 upregulated and 55 downregulated metabolites. Similarly, 89 DEMs were detected in CSF, including 24 upregulated and 65 downregulated metabolites. For the sham-operated groups, 79 DEMs were observed in serum between the HLJDD-treated sham group and the sham group (21 upregulated, 58 downregulated), while 91 DEMs were identified in CSF (22 upregulated, 69 downregulated) (Figures 7E–I).

### 3.8 Metabolic pathway analysis

**FIGURE 8:**
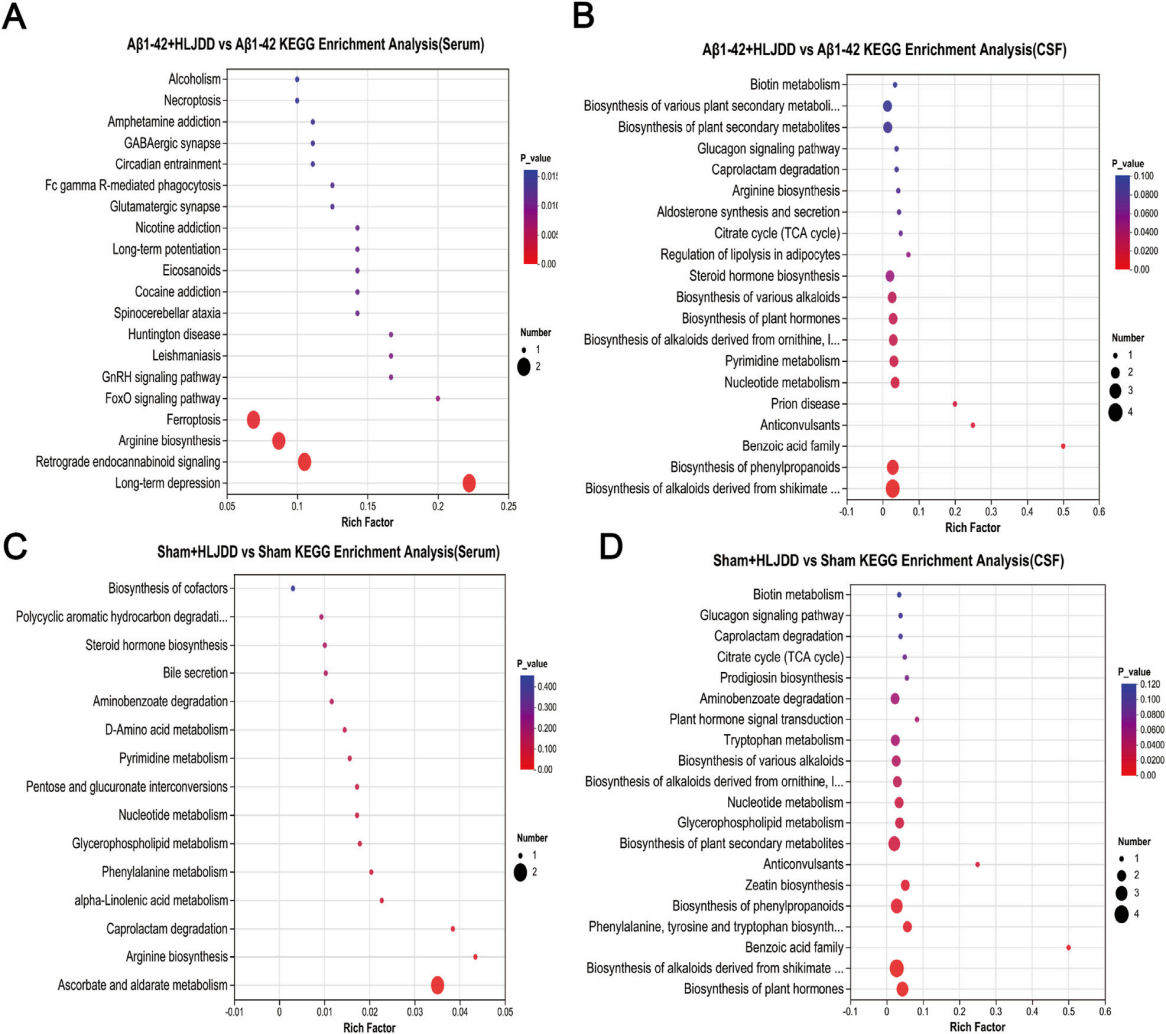
KEGG enrichment analysis of differential metabolites. **(A)** Aβ_1-42_ + HLJDD vs. Aβ_1-42_ KEGG enrichment analysis (serum), **(B)** Aβ_1-42_ + HLJDD vs. Aβ_1-42_ KEGG enrichment analysis (CSF), **(C)** Sham + HLJDD vs. Sham KEGG enrichment analysis (serum), **(D)** Sham + HLJDD vs. Sham KEGG enrichment analysis (CSF). Data are presented as mean ± SD (*n =* 6). *P*-value: t-test results to evaluate significant differences between two groups, with *P* < 0.05 considered significant.

To further elucidate the metabolic discrepancies underlying the differential effects of HLJDD between AD and normal rats, KEGG pathway enrichment analysis was performed on CSF and serum samples from the HLJDD-treated Aβ_1-42_ group and the Aβ_1-42_ group. The analysis revealed significant enrichment of various differentially expressed pathways in both serum and CSF, including long-term depression, retrograde endocannabinoid signaling, arginine biosynthesis, and ferroptosis. In CSF, notable alterations were observed in the biosynthesis of alkaloids derived from the shikimate pathway and the biosynthesis of phenylpropanoids. Arginine biosynthesis was identified as a commonly enriched pathway in both CSF and serum, suggesting its critical role in mediating the context-dependent therapeutic efficacy of HLJDD in AD and normal rats. Additionally, an analogous analysis between the HLJDD-treated sham group and the sham group identified co-enriched metabolic perturbations in pathways such as aminobenzoate degradation, nucleotide metabolism, caprolactam degradation, and glycerophospholipid metabolism across serum and CSF (Figure 8). These results suggest that HLJDD may disrupt these pathways in normal rats, potentially contributing to its organotoxic effects in non-pathological conditions.

### 3.9 Cluster analysis of differential metabolites

**FIGURE 9:**
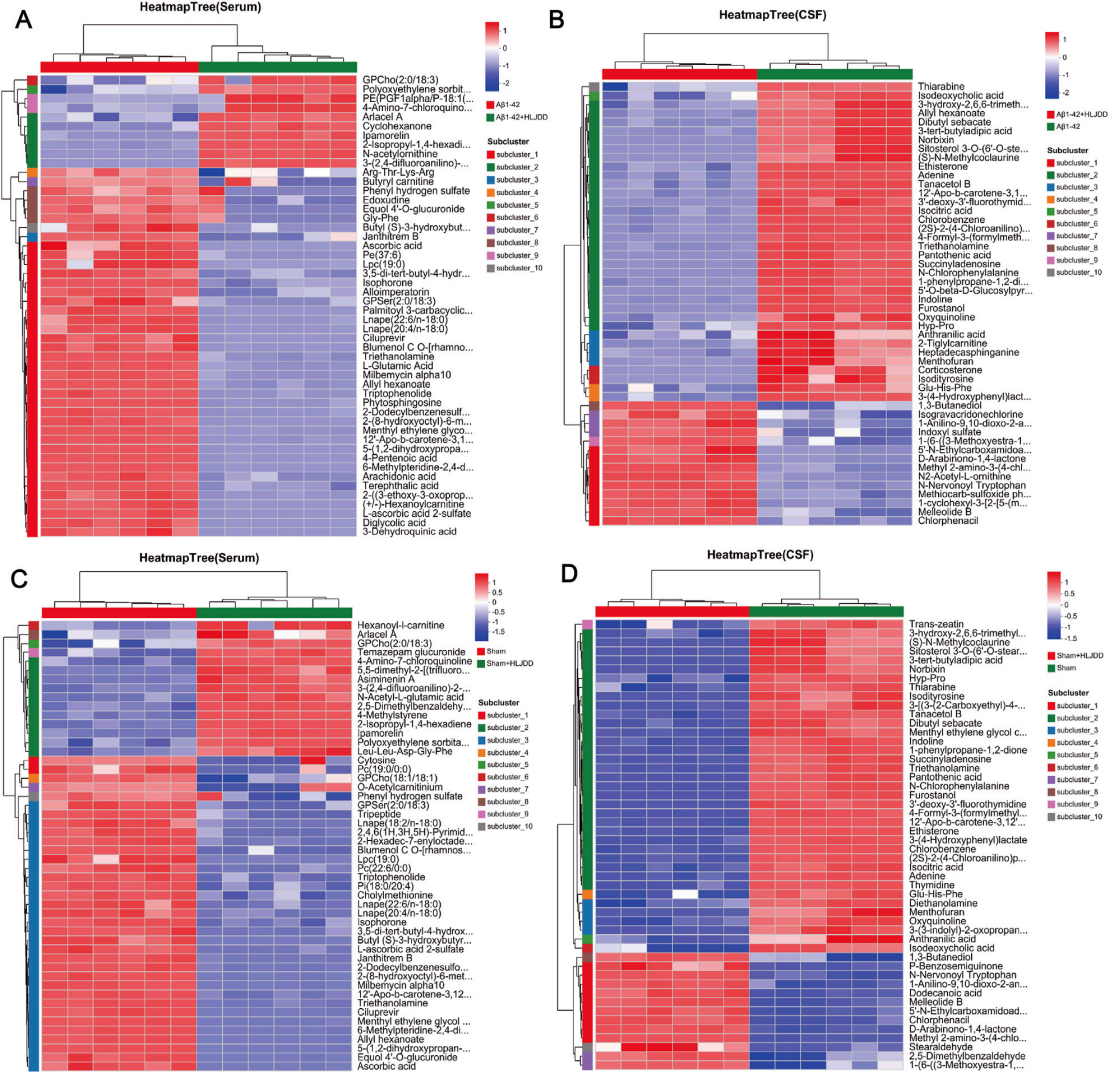
Hierarchical clustering diagram of differential metabolites. **(A)** Aβ_1-42_ vs. Aβ_1-42_ + HLJDD Heatmap (serum), **(B)** Aβ_1-42_ + HLJDD vs. Aβ_1-42_ Heatmap (CSF), **(C)** Sham vs. Sham + HLJDD Heatmap (serum), **(D)** Sham + HLJDD vs. Sham Heatmap (CSF). The color of each section represents the abundance value of each metabolite, calculated using the relative quantitation normalization method. The vertical axis represents significantly different metabolites, while the horizontal axis represents functional group information. Red and blue indicate upregulated and downregulated metabolites, respectively, in each sample (*n =* 6).

Cluster heatmap analysis of DEMs revealed distinct metabolic shifts between the experimental groups. In serum, N-acetylornithine and 3-methyl-2,5-furandione were significantly upregulated in the HLJDD-treated Aβ_1-42_ group compared to the Aβ_1-42_ group, while arachidonic acid, L-glutamic acid, and phytosphingosine were markedly downregulated. In CSF, N2-acetyl-L-ornithine and chlorphenacil levels were elevated, while triethanolamine and isocitric acid were reduced. For the sham-operated groups, serum analysis revealed upregulation of cyclohexanone and N-lauroylglycine in the HLJDD-treated sham group compared to the sham group, alongside downregulation of ascorbic acid, terephthalic acid, and cytosine. In CSF, benzamide and 1-anilino-9,10-dioxo-2-anthroic acid were upregulated, while adenine, thymidine, and aminocaproic acid were suppressed (Figure 9).

## 4 Discussion

To elucidate the mechanism of HLJDD in AD, an Aβ_1-42_-induced AD rat model was employed, a widely used approach for AD modeling due to its simplicity and reproducibility. It is important to note that this study utilized only male rats. This approach was chosen to eliminate potential confounds associated with the estrous cycle in females, thereby reducing biological variability and allowing for a more focused investigation into the core mechanisms of Aβ-induced toxicity and the effects of HLJDD. Histopathological evaluation (H&E and Nissl staining), IHC detection of Aβ deposition, and ELISA analysis of Aβ levels in CSF and serum revealed that HLJDD significantly alleviated brain injury, neuronal apoptosis, and Aβ deposition in AD rats. These findings align with the core pathological features of AD, in which Aβ neurotoxicity and subsequent neuronal apoptosis drive disease progression (Roda et al., 2022). IHC did not detect classic Aβ amyloid plaques, likely due to the transient nature of oligomers, which fail to reach the fibrillization threshold concentration necessary for plaque formation (Kramer et al., 2024). It is possible that the Aβ_1-42_ hippocampal CA1 injection model, being an acute and focal injury model, fails to replicate the characteristic progressive deposition of Aβ plaques seen in the AD process. Matrix metalloproteinases (MMPs), multifunctional endopeptidases, modulate AD pathogenesis by regulating Aβ metabolism, inflammatory cytokine secretion, and BBB integrity (Beroun et al., 2019; Hillmer et al., 2023; Rivera et al., 2019; Spampinato et al., 2017; Woo et al., 2008). The BBB also serves as a conduit for systemic inflammatory signals to the brain, creating an interplay between BBB dysfunction, Aβ accumulation, and neuroinflammation (Laurent et al., 2018; Milo et al., 2020; T. Zhang et al., 2015). HLJDD primarily restores BBB function by enhancing its structural integrity and suppressing local inflammation and adhesion molecule expression, rather than directly penetrating the brain (C. Chen et al., 2025). Our data further revealed that HLJDD administration significantly reduced MMP-2 and MMP-9 expression in the hippocampal CA1 region of AD rats, suggesting that its neuroprotective effects may involve BBB stabilization and anti-inflammatory regulation. In AD pathology, disrupted cerebral homeostasis and imbalanced inflammatory responses contribute to progressive neuronal loss and neuroinflammation (Kawahara et al., 2012; Minghetti et al., 2005; Sheng et al., 1996; Shepherd et al., 2000; Webers et al., 2020; Wynn et al., 2011). Notably, HLJDD treatment significantly reduced inflammatory cytokine levels in both CSF and serum of AD rats, supporting its dual anti-inflammatory actions in both peripheral and central compartments. Collectively, HLJDD demonstrates potential neuroprotective effects against AD through mechanisms involving Aβ deposition reduction, neuronal apoptosis inhibition, BBB integrity improvement, and suppression of peripheral and central inflammatory cascades. These findings provide critical insights into the pharmacological mechanisms of HLJDD and its therapeutic potential for AD. Our study identified 23 metabolites in HLJDD, primarily comprising flavonoids, alkaloids, and iridoids. It was demonstrated that the formula exerts neuroprotective effects primarily through the core representative component berberine (Y. Yang et al., 2013; Durairajan et al., 2012), in conjunction with alkaloids including jatrorrhizine and palmatine. These effects are mediated mainly by the alleviation of oxidative stress and suppression of neuroinflammation, with synergistic contributions from other active constituents such as wogonoside, baicalin, and geniposide (Cao et al., 2023).

Although HLJDD demonstrates significant neuroprotective effects in AD rats, its potential toxicity and underlying mechanisms in normal rats warrant critical evaluation. Under physiological conditions, the balance between pro-inflammatory and anti-inflammatory cytokines maintains systemic homeostasis (Anisman, 2004). However, HLJDD administration in normal rats paradoxically increased inflammatory cytokine levels in both CSF and serum, accompanied by heightened neuronal apoptosis, indicating that HLJDD may disrupt this balance, triggering peripheral and central inflammation along with partial neuronal damage. Furthermore, MMP-9 expression was significantly upregulated in this group. Given the established correlation between MMP-9 overexpression and exacerbated neuroinflammation, this change is hypothesized to further compromise BBB integrity in normal rats. However, this interpretation remains speculative due to the lack of key functional evidence, such as measurements of BBB permeability using Evans Blue dye or IgG immunohistochemistry. Therefore, future studies incorporating these assays are essential to validate this hypothesis. Previous studies have identified the liver as a primary target organ for HLJDD components (Yamano et al., 1988; Ma et al., 2010), with Coptidis Rhizoma (a key HLJDD metabolites) specifically linked to hepatotoxicity (Ma et al., 2010). Additionally, the kidneys, with their high vascularity and energy demands, are susceptible to drug-induced injury via systemic circulation (Griffin et al., 2019). Histopathological analysis using H&E staining revealed varying degrees of damage in the liver, kidneys, and large intestine of normal rats treated with HLJDD. Notably, oral administration of bitter metabolites is known to activate gastrointestinal protective mechanisms (e.g., diarrhea) (Meyerhof et al., 2010), while prolonged use of Coptidis Rhizoma has been shown to suppress intestinal motility and impair colonic barrier function in mice (Wu et al., 2025). As a bitter botanical drugs, long-term HLJDD use may induce adverse gastrointestinal effects. Consistent with these findings, HLJDD-treated sham-operated rats exhibited perianal soiling, loose stools, and large intestinal lesions. While histopathological observations revealed tissue alterations in the liver, kidneys, and large intestine of normal rats following HLJDD administration, the absence of supporting serum biochemical parameters (specifically ALT, AST, BUN, and creatinine) precludes a definitive conclusion on organ toxicity. Therefore, the present findings cannot establish but may hint at potential toxicological implications. These preliminary outcomes nonetheless provide crucial initial evidence, underscoring a need for more comprehensive safety evaluations that integrate both histopathological and serological data in future studies.

Drug toxicity and pharmacokinetics vary significantly depending on an individual’s physiological condition. For example, the toxicity of digoxin differs markedly between healthy individuals and those with heart failure. Metabolomic analysis revealed that the therapeutic effects of HLJDD in AD are linked to multiple metabolic pathways, including ferroptosis, the biosynthesis of alkaloids derived from the shikimate pathway, and arginine biosynthesis. Notably, the arginine biosynthesis pathway was co-enriched in both serum and CSF, highlighting its critical role in AD pathogenesis (Horgusluoglu et al., 2022). The ferroptosis pathway is particularly noteworthy, showing significant correlations with key metabolites such as arachidonic acid and L-glutamic acid. Ferroptosis, an iron-dependent form of regulated cell death, may exacerbate neuronal damage in AD through glutamic acid excitotoxicity. L-glutamic acid, the primary excitatory neurotransmitter in the central nervous system, is pathologically elevated in AD. This elevation not only promotes cerebral tissue injury and neuronal apoptosis (Belousov et al., 2012) but also disrupts BBB integrity (Gruenbaum et al., 2022), accelerating AD progression through impaired clearance mechanisms (Bell et al., 2003; Tatara et al., 2023). Additionally, arachidonic acid, a polyunsaturated fatty acid critical for microglial activation, exacerbates neuroinflammation and BBB dysfunction under pathological conditions (Xu et al., 2024; Yagami et al., 2018; Ziegler et al., 2016). Baicalin, a bioactive component of HLJDD, modulates arachidonic acid metabolism (S. Chen et al., 2019), thereby reducing inflammatory infiltration and brain injury (Hwang et al., 2002). Consistent with this, HLJDD-treated Aβ_1-42_ -induced AD rats exhibited reduced serum levels of L-glutamic acid and arachidonic acid, alleviated CSF and serum inflammation, and restored BBB integrity. Moreover, abnormal elevations of isocitrate and citrate in patients with AD reflect dysregulated energy metabolism and cell cycle control (Bubber et al., 2005; van der Velpen et al., 2019). These metabolic disturbances aggravate neuroinflammation and neuronal apoptosis, ultimately impairing cognitive function (Hotamisligil, 2006). HLJDD intervention downregulated the levels of arachidonic acid, L-glutamic acid, and isocitric acid while upregulating N-acetylornithine and N2-acetyl-L-ornithine in AD rats. These findings suggest that HLJDD may ameliorate AD pathology by modulating arginine biosynthesis, which is associated with reduced apoptosis, diminished peripheral and central inflammation, and restored BBB integrity. Significant alterations in multiple metabolic pathways, including nucleotide metabolism, glycerophospholipid metabolism, and caprolactam degradation, were also observed in the CSF and serum of normal rats treated with HLJDD.

Disruption of nucleotide metabolism can impair mitochondrial function in the kidneys, compromise cellular barrier integrity, and directly contribute to renal injury, promoting inflammation and fibrosis (Ralto et al., 2020). Among its key components, adenine and cytosine are closely linked to inflammatory regulation. Adenine is converted to adenosine monophosphate (AMP) via the salvage pathway (Dias et al., 2023). As an endogenous signaling molecule, adenosine exerts cytoprotective and anti-inflammatory effects under pathological conditions through upregulation of ectonucleotidase activity (Li et al., 2020). Cytosine, in contrast, modulates cytokine expression and contributes to inflammation (Meng et al., 2021). In this study, reduced levels of adenine and cytosine were observed in HLJDD-treated sham rats, suggesting metabolic suppression mythat may exacerbate inflammatory responses. Restoring mitochondrial membrane potential can reduce pro-inflammatory cytokine release and excessive MMP-9 expression (Sun et al., 2021). In this study, reduced levels of adenine and cytosine were observed in HLJDD-treated sham rats, suggesting metabolic suppression that may exacerbate inflammatory responses. Restoring mitochondrial membrane potential can reduce pro-inflammatory cytokine release and excessive MMP-9 expression (Brouns et al., 2010; Rochfort and Cummins, 2015). In line with this, HLJDD-treated sham rats exhibited peripheral and central inflammation, BBB dysfunction, and neuronal apoptosis, implying that their pathological changes may be related to metabolism imbalance. Notably, the most significantly altered pathway appeared to involve ascorbate and aldarate metabolism, characterized by reduced levels of ascorbic acid. Ascorbic acid enhances the activity of hepatic UDP-glucuronosyltransferase, a critical enzyme for liver function, and its deficiency exacerbates liver injury (Arendt and Allard, 2011). Previous studies have reported the potential hepatotoxicity of geniposide, a metabolite of HLJDD (Yamano et al., 1990). Confirming this, UPLC-Q-Exactive Orbitrap HRMS analysis in the current study identified geniposide within the HLJDD preparation. However, the hepatotoxic mechanism of HLJDD as a complex formula is likely multifactorial and cannot be solely attributed to geniposide. The results suggest that HLJDD-induced liver injury could be related to geniposide but might also involve disturbances in the ascorbate metabolic pathway. Non-targeted metabolomic analysis in this study revealed that HLJDD induces significant metabolic perturbations in both AD and normal rats, albeit with fundamental differences in the direction and magnitude of its effects. Notably, our KEGG pathway analysis highlighted several pathways exhibiting significant alterations in both animal groups. These included the Phenylalanine, tyrosine and tryptophan biosynthesis, Biosynthesis of alkaloids derived from shikimate pathway, and Protein digestion and absorption pathways in the CSF; as well as the Choline metabolism in cancer, Glycerophospholipid metabolism, Linoleic acid metabolism, and Retrograde endocannabinoid signaling pathways in the serum. These findings strongly suggest that the aforementioned pathways may represent critical targets for the bidirectional regulatory effects of HLJDD. It is important to note, however, that this conclusion remains preliminary and is derived primarily from metabolomic data. Further validation using molecular biology experiments is therefore warranted. We hope that these insights will provide valuable hypotheses and a solid foundation for subsequent research in the field.

## 5 Limitations

This study also has several aspects that could be further improved. First, although we assessed changes in MMP-2 and MMP-9 levels via immunohistochemistry, direct functional assays such as the Evans blue extravasation test were not performed to validate actual alterations in blood–brain barrier permeability. Second, while the observed metabolomic shifts suggest potential underlying mechanisms, the causal relationship between these changes and the observed effects remains unclear due to the lack of functional validation. Future studies should employ targeted metabolomics to identify key metabolites and further verify their functional roles. Third, the study did not include cognitive or behavioral assessments. As the primary focus was on pathological and biomarker differences between AD and normal rats, the absence of functional behavioral evaluation limits the interpretation of functional recovery, which should be addressed in future research. Lastly, although this single-dose study confirmed the toxic risk of HLJDD at the administered dose and revealed histopathological alterations in organs, the toxicological implications of these findings require more comprehensive evaluation incorporating serum biochemical parameters. Moreover, whether these toxic effects are reversible remains undetermined and should be clarified through subsequent continuous monitoring. Nevertheless, these findings collectively highlight the importance of closely monitoring organ functional safety in the clinical application of HLJDD.

## 6 Conclusion

In AD rats, HLJDD reduced Aβ deposition, enhanced neuronal survival, ameliorated systemic inflammation, improved BBB integrity, and attenuated cerebral, renal, and gastric injuries without causing organ damage, indicating its safety. These effects were mediated by modulation of the arginine biosynthesis pathway. In contrast, in healthy rats, HLJDD induced systemic inflammation, compromised neuronal and BBB integrity, and caused hepatic, renal, and colonic injuries. These differential effects suggest the possible involvement of metabolic pathways, including nucleotide metabolism, aminobenzoate degradation, glycerophospholipid metabolism, and caprolactam degradation. Collectively, these findings highlight that HLJDD exhibits context-dependent pharmacology, necessitating a precise mechanistic understanding for safe clinical application.

## Data Availability

Source data have been deposited to the EMBL-EBI MetaboLights database with the identifier MTBLS13256. Further inquiries can be directed to the corresponding author.
